# Erythema *elevatum diutinum* as a first clinical manifestation for diagnosing HIV infection: case history

**DOI:** 10.1590/S1516-31802005000400009

**Published:** 2005-07-07

**Authors:** Patrícia Accioni Rover, Caroline Bittencourt, Mariana Pimenta Discacciati, Mariana Colombini Zaniboni, Lúcia Helena de Fávaro Arruda, Maria Letícia Cintra

**Keywords:** Skin diseases, HIV, Vasculitis, Streptococcal infections, Dapsone, Dermatopatias, HIV, Vasculite, Infecções estreptocócicas, Dapsona

## Abstract

**CONTEXT:**

Erythema *elevatum diutinum* is a chronic and rare dermatosis that is considered to be a variant of leukocytoclastic vasculitis. It is probably mediated by immune complexes. It is generally associated with autoimmune, neoplastic and infectious processes. Recently, it has been added to the group of specific dermatoses that are associated with HIV.

**CASE REPORT:**

We report on the case of a patient who had erythema *elevatum diutinum*as the first clinical evidence for diagnosing HIV infection. Dapsone was used in the treatment of this patient, and partial regression of the lesions was achieved within 15 days, even before antiretroviral therapy was prescribed.

**CONCLUSION:**

When there is a diagnosis of erythema elevatum diutinum, HIV infection should be investigated, especially in atypical and exacerbated clinical manifestations.

## RESUMO

**CONTEXTO:**

O eritema *elevatum diutinum* é uma dermatose crônica, rara, variante clínica da vasculite leucocitoclástica, provavelmente mediada por imunocomplexos. Está sendo incluído no grupo das dermatoses específicas associadas à infecção pelo HIV. Em geral, associa-se a outros processos infecciosos, auto-imunes e neoplásicos.

**RELATO DE CASO:**

Relatamos o caso de um paciente em que a manifestação cutânea de eritema *elevatum diutinum* foi a primeira evidência clínica para diagnóstico da infecção pelo HIV. O tratamento foi feito com dapsona e se obteve regressão parcial das lesões após 15 dias, mesmo antes de o esquema antiretroviral ser prescrito.

**CONCLUSÃO:**

Frente ao diagnóstico de eritema *elevatum diutinum*, deve-se investigar a infecção pelo HIV, principalmente nas apresentações clínicas atípicas e exacerbadas.

## INTRODUCTION

Erythema elevatum diutinum (EED) is a chronic and rare dermatosis that is considered to be a variant of leukocytoclastic vasculitis. The clinical manifestations are papules, plaques or nodules, which vary in coloration from reddish to purple, light brown and sometimes yellowish. The lesions are persistent and symmetrically distributed on extensor surfaces, particularly in the joints of the extremities. Such patients may present arthralgia, itchiness and pain, with rare systemic involvement. In general, their overall condition is not compromised.^1,2^ The disease affects both sexes, and is more frequent between the ages of 30 and 60 years.^1^ The histopathological analysis of acute lesions is characterized by the presence of leukocytoclastic vasculitis, with marked infiltration of polymorphonuclear neutrophils and deposition of fibrinoid material. Over the course of the disease, collagen is deposited around the vessels and, occasionally, cholesterol crystals are detected.^3^

The etiology of EED remains unknown. It is probable that immune complex deposition occurs in vessel walls,^1^ secondary to streptococcal infections^4^ and hematological^3,5^ and autoimmune diseases.^6,7^ Recently, cases of association with HIV^8^ have been described. We report on the case of a patient in whom cutaneous manifestation of erythema elevatum diutinum was the first clinical evidence for diagnosing HIV infection.

## CASE REPORT

**Figure 1:**
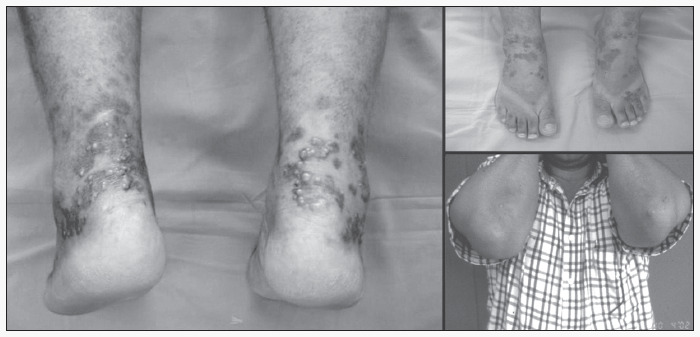
Violet-colored confluent erythematous papules on the feet and ankles of a man with HIV infection.

A 48-year old male Caucasian patient, born and residing in Campinas, had a history of hyperchromic macules, bilaterally located in the medial and lateral malleolar zone of the ankles and on the heels and dorsal aspect of the feet. Subsequently, the lesions evolved with the formation of violet-colored erythematous papules that were confluent in plaques with a light brown surface (Figure 1).

In his personal history, sexual promiscuity had been reported, although not drug addiction. Laboratory tests showed discrete leukopenia, while his hepatic and renal function and protein and immunoglobulin electrophoresis presented no abnormalities. The blood test was negative for hepatitis B and C, and positive for HIV.

**Figure. 2:**
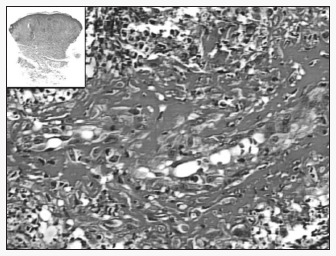
Dense infiltrate of neutrophils with fibrin deposition and nuclear fragments within superficial and deep blood vessel walls (hematoxylin and eosin; original magnification 400 X; inset 25 X), in a case of erythema *elevatum diutinum* in a man with HIV infection.

The histopathological examination revealed blood vessels with rather thickened walls that were dissociated by fibrin, with neutrophils that were often fragmented, i.e. necrotizing leukocytoclastic vasculitis (Figure 2).

Dapsone at a dose of 100 mg/day was prescribed. Fifteen days later, partial regression of the lesions was achieved, without any antiretroviral therapy yet prescribed. One month after diagnosing HIV infection, the patient presented a neurological condition of right-side motor impairment. Neurotoxoplasmosis of the central nervous system was diagnosed by means of computed tomography of the head, and the patient was hospitalized for 15 days for treatment. On this occasion, antiretroviral therapy was begun. The patient was no longer using dapsone and dermatological examination showed only residual lesions. Today, one-and-a-half years after the HIVpositive blood test and the beginning of antiretroviral therapy, the patient has not had any recurrence of the skin lesions.

## DISCUSSION

There is still controversy about the etiology of EED. Nevertheless, the most widely accepted theory is that previous and repeated exposure to bacterial infections, particularly streptococcal ones, may trigger an immunological reaction that culminates in an outbreak of skin lesions.^9,10^ It is believed that the immune complex is deposited in vessel walls and subsequently phagocytized by the neutrophils.^1^

EED has been described in association with numerous hematological abnormalities, especially myelodysplasia, myeloproliferative alterations, multiple myeloma,^3,11^ cryoglobulinemia^12^ and immunoglobulin G (IgG) or immunoglobulin A (IgA) paraproteinemias.^13-15^ There have also been reports of associations with rheumatic arthritis,^3,16,17^ prostate carcinoma,^3,18^ testicular lymphoma,^3^ celiac disease,^19^ Crohn's disease,^20^ relapsing polychondritis^21^ and, more recently, with HIV infection.^22-25^ Among young males, exacerbated clinical states with nodular lesions seem to be correlated with HIV infection, in contrast with the clinical picture observed for the general population.^24,25^

The EED skin lesions of this patient allowed the detection of HIV infection through laboratory investigation. There was no history of streptococcal infection, hematological disorders or autoimmune disease. Reports of an association between EED and HIV have only sporadically been published. Over the last few years, eleven EED cases have been described in patients who already knew they were HIV-positive. It is believed that such an association results from the HIV antigen-antibody interaction, which causes direct damage to vessel walls. It is also supposed that the immunosuppression caused by HIV predisposes towards infection by other agents that trigger an antigenic stimulus for the development of EED.^8^

Consequently, even though the association of EED with HIV infection is infrequent, laboratory investigation for this virus should be requested in conventional cases, and especially in cases of atypical and exacerbated clinical manifestations. In addition, antiretroviral therapy should be introduced in these cases, in association with dapsone, which is the drug of choice for the treatment of EED.^25,26^
